# Lung involvement at presentation predicts disease activity and permanent organ damage at 6, 12 and 24 months follow - up in ANCA - associated vasculitis

**DOI:** 10.1186/1471-2172-15-20

**Published:** 2014-05-27

**Authors:** Tidi M Hassan, Astrid S Hassan, Ann Igoe, Mark Logan, Cedric Gunaratnam, Noel G McElvaney, Shane J O’Neill

**Affiliations:** 1 Department of Respiratory, Beaumont Hospital, Beaumont Road, Dublin 9, Ireland

**Keywords:** ANCA, Vasculitis, Pulmonary hemorrhage

## Abstract

**Background:**

Antineutrophilic cytoplasmic antibody (ANCA)-associated vasculitis (AAV) may present with pulmonary involvement ranging from mild to life-threatening disease such as diffuse alveolar hemorrhage. There is a paucity of information regarding morbidity outcomes for AAV subjects presenting with lung involvement. This study determines the relationship between disease activity and damage in these subjects using the Birmingham Vasculitis Activity Score v 3 (BVAS 3) and Vasculitis Damage Index (VDI) respectively.

**Results:**

151 patients with AAV were included with 59 presenting initially with pulmonary involvement. The initial BVAS scores recorded at time of diagnosis were positively correlated with the final VDI scores at 24 months (p < 0.0001, rs = 0.5871). No differences between BVAS and VDI scores were seen for both groups, however in the lung-involvement group only, BVAS scores were significantly higher at 6, 12 and 24 months whilst the VDI scores were significantly higher at 12 and 24 months. Subjects presenting with pulmonary involvement had an increased likelihood for cardiovascular (OR 1.31, 95% CI 0.89, 1.54; p = 0.032) and renal (OR 1.32, 95% CI 1.22, 1.39; p = 0.005) involvement. Subjects presenting with lung involvement with granulomatosis with polyangiitis and microscopic polyangiitis had 24-month VDI scores that were significantly higher (p = 0.027, p = 0.045), and more likely to develop pulmonary fibrosis (OR 1.79, 95% CI 1.48, 2.12; p < 0.001).

**Conclusion:**

AAV subjects with lung involvement at presentation had a higher disease activity and damage scores at 6, 12 and 24 months follow-up representing a considerable burden of disease despite improvement in overall survival due to the introduction of immunosuppressive therapy.

## Background

Antineutrophilic cytoplasmic antibody (ANCA)-associated vasculitis (AAV) is a potentially life-threatening condition [1]. The introduction of immunosuppressive therapy has transformed this condition from an outcome of 80% mortality at one year to a survival of 55% and 75% in microscopic polyangiitis (MPA) and granulomatosis with polyangiitis (GPA) respectively at 10 years [2,3]. Very active disease at presentation, continuous disease activity, relapse and drug toxicity may cause irreversible damage leading to considerable burden of disease. Therefore, studies to stratify prognostic indicators of AAV and identify high-risk subjects are crucial [4,5].

Pulmonary involvement (PI) is a major characteristic feature of both GPA and Churg-Strauss syndrome (CS), but less common in MPA [6]. Diffuse alveolar hemorrhage (DAH) is one of the most severe pulmonary manifestations. The prognostic value of PI in AAV remains controversial. One study reported that DAH at presentation in MPA was associated with a relative risk (RR) of 8.65 for mortality [7]. Reinhold-Keller reported that PI was associated with a RR of 3.75 for mortality in GPA [8]. Another study showed that the early death risk was 15 times higher in subjects presenting with cough in GPA [9]. However, a more recent study concluded that DAH alone is not predictive of a poor prognosis [10]. Meanwhile, the revised Five-Factor Score (FFS), a score designed to predict survival concluded that ENT manifestations were associated with favorable outcomes in GPA [11].

Although survival rates have improved, disease activity and subsequent burden of disease remain important outcomes in assessing the overall well being of AAV individuals. The objective of this study was to determine the relationship between disease activity and damage in subjects presenting with PI and lung involvement (LI) using the Birmingham Vasculitis Activity Score v.3 (BVAS3) and Vasculitis Damage Index (VDI) respectively.

## Methods

### Patient selection

We enrolled 151 subjects with AAV including GPA, MPA and CS into this study from a single tertiary center in Dublin, Ireland. The study was discussed with Beaumont Hospital Research Ethics Committee who waived the need for ethical approval. The diagnoses satisfied the American College of Rheumatology (ACR) criteria and/or the Chapel Hill Consensus nomenclature [12]. These adult subjects were recruited from either the pulmonology, rheumatology, nephrology or dermatology clinic from July 2008 to June 2012. No written informed consent was obtained as the BVAS and VDI scores were performed routinely.

### Data collection

Subjects’ demographics, initial symptoms, date of diagnosis, physical examination findings, ANCA levels, blood biochemistry, diagnostic imaging, relapses and treatment regimens were recorded. Relapses are defined within the newly developed clinical finding of GPA and adapted to the diseases according to BVAS definition [6]. Cytoplasmic or proteinase 3(c-/PR3) and perinuclear or myeloperoxidase (p-/MPO) ANCA were measured at the same time point using ELISA. Biopsies including renal, skin, nasal and pulmonary showing capillaritis confirmed the AAV diagnosis, when needed.

All subjects with PI at initial presentation were followed up in the pulmonology clinic with pulmonary function test and chest radiograph at 3-,6-, 12-month and 2-year follow-up. The BVAS3 was calculated at 0-, 6-, 12-month and 2-year follow-up while the VDI was calculated at 6-, 12-month and 2-year. Both BVAS3 and VDI scores between 0- and 6-months of ANCA-vasculitis diagnosed before June 2009 were scored retrospectively from hospital charts and radiological investigations. BVAS3 and VDI were recorded at 12-month and 2-year prospectively. For subjects diagnosed after June 2009 (n = 94), data were collected prospectively. Intraobserver reliability of BVAS and VDI was examined in all subjects with a second assessment by the same observer within the same week of the first assessment. Interobserver reliability was evaluated in 20 subjects independently assessed by two observers on the same day. We also examined the chest radiograph or CT thorax performed within two weeks of initial clinical presentation. All subjects had at least one chest radiograph within a month of their initial presentation.

### Statistical analysis

All statistical analyses were performed using GraphPad Prism 5 software package (San Diego, CA). Correlation of BVAS3 and VDI scores was analyzed by the Spearman correlation test. Differences were analyzed by the Student *t*-test (non-parametric, one-tailed; Mann Whitney) as appropriate. In multivariate analyses, the prevalence odds ratio (OR) with 95% confidence interval (CI) for the association between VDI and clinical presentation was estimated using logistic regression.

## Results

### Patient demographics

The dataset included 151 subjects; 6 subjects had data which was unattainable for at least 1 variable. 51 with GPA, 58 with MPA, 9 with CS and 21 with polyarteritis nodosa (PAN) are included as the majority of subjects in this analysis. 2 subjects were diagnosed with idiopathic pauci-immune pulmonary capillaritis (IPIPC). Table 1 shows the main characteristics for the overall study population with and without PI. Mean age at diagnosis was 45 ± 11.2 years. Mean (SD) follow-up was 2.22 years and there were 8 deaths. Subjects that did not present with PI had a significantly higher rate of relapse compared to those who did (p < 0.05).

**Table 1:** Baseline demographic and clinical characteristics with anti-neutrophil cytoplasmic antibody (ANCA) associated vasculitis (AAV), within subgroups with pulmonary and non-pulmonary involvement at initial presentation of AAV

**Patient characteristics**	**Pulmonary involvement**	**Non-pulmonary involvement**
	**(n = 59)**	**(n = 92)**
Age, mean +/- SD years	43 +/- 12.9	49 +/1 10.1
Male sex, n (%)	29 (49)	49 (53)
AAV diagnosis, n (%)		
GPA	38 (64.4)	13 (14.1)
MPA	9 (15.3)	49 (53.3)
CS	5 (8.5)	4 (4.3)
PAN	4 (6.7)	17 (18.5)
Others	3 (5.1)	9 (9.8)
Mean serum creatinine, uM (SD)	176 +/- 139	251 +/- 161
ANCA positivity, n (%)		
MPO ANCA	17 (29)	58 (63)
PR3 ANCA	45 (76)	41 (45)
Relapses	12 (20.3)	25 (27)
1-Relapse	7 (11.8)	15 (16.3)
2-Relapse	1 (1.7)	3 (3.3)
3-Relapse	1 (1.7)	3 (3.3)
> 3-relapse	3 (5.1)	4 (4.3)
Immunosuppressive therapy within 2 months of intial presentation, n (%)		
Cyclophosphamide	26 (44)	59 (62)
Glucocorticoids	29 (49)	65 (70)
Azathioprine	3 (5)	6 (6)
Methotrexate	8 (14)	4 (4)
Mycophenolate Mofetil	0 (0)	0 (0)
Mean follow-up, years (SD)	2.1 (2.9)	2.24 (2.89)
Deaths, n	4 (6.8)	4 (4.3)

PI at initial presentation was present in 59 subjects. 14 subjects had overlapping pulmonary diseases involving either endobronchial or parenchymal disease at initial presentation. Subjects with PI at initial presentation exhibit higher mortality than non-PI subjects (6.8 vs 4.3) however this was not statistically significant. All 8 subjects who died on this study had a VDI of >4. 4 subjects with active vasculitis died due to sepsis and multi-organ failure. The other 4 deaths were due to cardiac arrest from active renal disease, a myocardial infarction due to long-standing coronary artery disease and 2 died from respiratory failure secondary to bronchopneumonia. No subjects died due to primary active disease in the lungs. Malignancy was not observed in any patient.

### Serum blood test at initial presentation

The mean (SD) serum creatinine was 204 ± 145 uM at time of diagnosis. The mean (SD) serum creatinine was higher in subjects with non-PI at 251 ± 161 uM compared to 176 ± 139 in subjects with PI. 14 subjects in the PI group were diagnosed as having pulmonary-renal syndromes. ANCA levels were positive in all subjects. PR3 ANCA was the major ANCA for the PI group while MPO ANCA was more common with no PI. 58% of subjects had PR3 ANCA of more than 20 U/ml in the PI group compared to 27% in the non-PI group. High titres of MPO ANCA of more than 20 U/ml were predominantly seen in the group with non-PI.

### BVAS and VDI at 0, 6, 12 and 24 months

**Figure 1:**
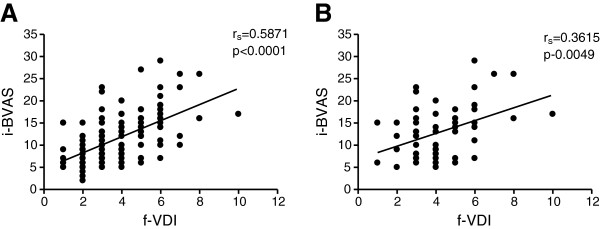
**Association of BVAS scores at initial presentation (i-BVAS) with VDI scores at 2 years follow-up (f-VDI) in (A) non-pulmonary and (B) pulmonary involvement groups.** Spearman’s correlation coefficient *r*_*s*_ and *p*-value [95% CI] included in the analysis.

**Figure 2:**
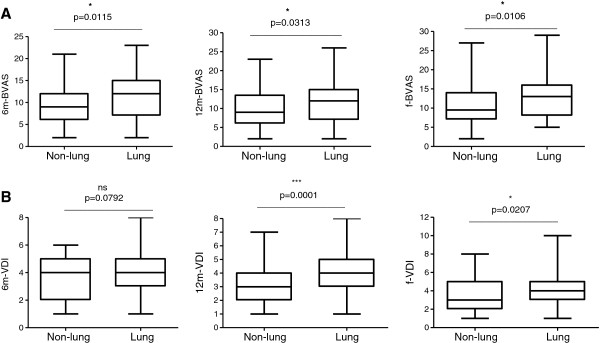
**(A) BVAS 3 and (B) VDI scores at 6-, 12- and 24 (f) months follow-up in non-lung and lung involvement groups.** Data were compared by non-parametric *t*-test (Mann Whitney U) with a significance *p*-value cut-off of 0.05 (*p < 0.05, **p < 0.01, ***p < 0.001).

Both the BVAS3 and VDI scores at initial presentation did not correlate with the number of subsequent relapses and the type of immunosuppressive therapy employed within 2 months of initial presentation. The initial BVAS scores recorded at time of diagnosis were found to be positively correlated with the final VDI scores at 24 months (p < 0.0001, r_s_ = 0.5871) (Figure 1). This was also observed in subjects with PI only (p = 0.0049, r_s_ = 0. 3615). Whilst there were no differences between BVAS and VDI scores for both patient groups, differences were seen when the PI subjects were subcategorized into LI group, accounting for subjects that only had pathology in either the lung parenchyma, airways, pleura and pulmonary arteries. BVAS3 scores were significantly higher in subjects with LI at 6, 12 and 24 months of follow-up (Figure 2A). The initial BVAS scores were also significantly higher in subjects presenting with LI (Additional file 1: Figure S1). The VDI recorded at 6, 12 and 24 months were higher in subjects with initial LI with differences significant for month 12 and 24 (Figure 2B).

At baseline, subjects with PI were more likely to have general systemic features and musculoskeletal involvement compared to those without PI (OR 1.32; 95% CI 1.11,1,59; p = 0.032, OR 1.62; 95% CI 1.15,2.31; p = <0.001 respectively) (Table 2). Subjects presenting with PI had an increased likelihood of cardiovascular (OR 1.31, 95% CI 0.89,1.54; p = 0.032) and renal (OR 1.32, 95% CI 1.22, 1.39, p = 0.005) involvement. Both groups had an average of 3 organ systems involved at the time of diagnosis.

**Table 2:** Association between subjects with lung invovlement and the presence of other vasculitis manifestation as per BVAS 3 categories within 2 years of follow-up adjusted for age and gender

**Organ involvement**	**OR (95% CI)**	***P*-value**
General	1.32 (1.11, 1.59)	0.032
Skin/Mucous membrane	0.96 (0.91, 1.08)	0.280
Ocular	0.81 (0.69, 1.21)	0.637
Musculoskeletal	1.62 (1.15, 2.31)	<0.001
Cardiovascular	1.31 (0.89, 1.54)	0.032
Peripheral vascular disease	1.01 (0.9, 1.28)	0.806
Gastrointestinal	0.99 (0.98, 1.13)	0.118
Renal	1.32 (1.22, 1.39)	0.005
Neuropsychiatric	1.00 (0.89, 1.11)	0.074
Other	1.14 (1.09, 1.19)	0.549

### Types of pulmonary manifestations and AAV phenotype

**Figure 3:**
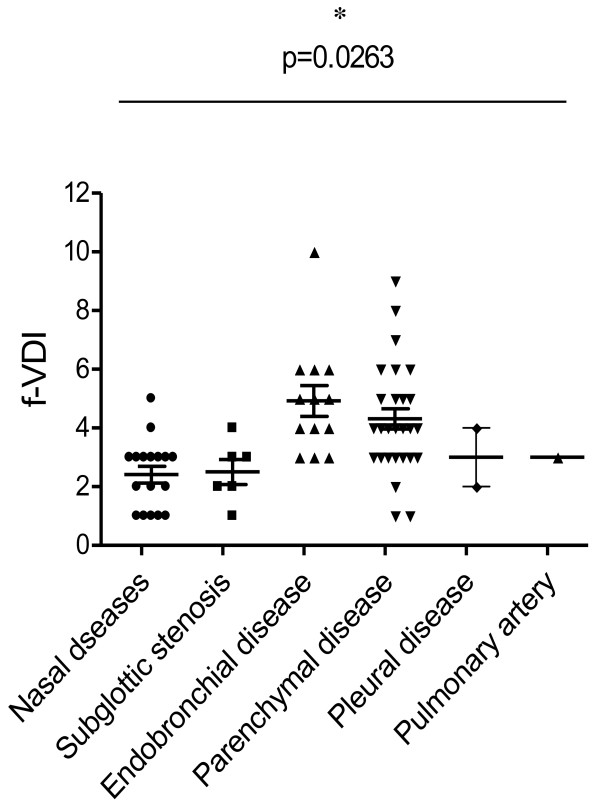
**VDI scores at 2-year follow-up (f-VDI) in subjects initially presenting with different pulmonary manifestations (which may overlap).** Data are represented as mean ± SEM. A three-way ANOVA test was used to calculate differences between the groups with a significance *p*-value cut-off of 0.05 (*p < 0.05, **p < 0.01, ***p < 0.001).

17 and 6 subjects presented with nasal diseases and subglottic stenosis respectively (Figure 3). 13 subjects presented with endobronchial diseases including airway obstruction, segmental bronchial wall thickening, tracheal wall abnormalities and bronchiectasis. 1 patient had ulcerating upper airway disease. 4 subjects who developed chronic asthma were both diagnosed with CS. 2 subjects presented with pleural nodules on chest radiograph. Both had overlapping presentation with nasal diseases and parenchymal disease. 29 subjects presented with parenchymal disease in which 12 presented with DAH. 11 presented with pulmonary nodules whilst 9 presented with cavitating lesions with overlapping features. These subjects were exclusively diagnosed with either GPA or MPA.

**Figure 4:**
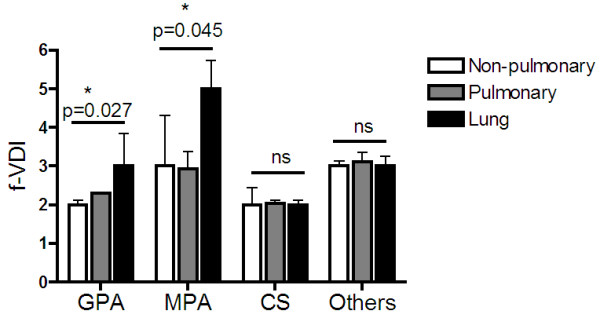
**VDI scores at 2-year follow-up (f-VDI) in subjects presenting initially with lung, pulmonary and non-pulmonary manifestations diagnosed with different AAV phenotype.** Data are represented as mean ± SEM. A three-way ANOVA test was used to calculate differences between the groups with a significance *p*-value cut-off of 0.05 (*p < 0.05, **p < 0.01, ***p < 0.001).

When LI subjects were categorized based on their diagnoses, GPA and MPA had a final VDI score that was significantly higher than their non-LI and PI counterparts with the same diagnosis (Figure 4). At 2-year follow-up, subjects with LI were also more likely to develop pulmonary fibrosis (OR 1.79; 95% CI 1.48, 2.12; p < 0.001) and impaired lung function (OR 1.42; 95% CI 1.27, 1.84; p = 0.045) (Table 3). 4 out of 12 subjects presenting with DAH developed pulmonary fibrosis. When the different lung function parameters were assessed, only diffusing capacity for carbon monoxide corrected to alveolar volume (DLCO/VA) was significantly lower than subjects in the non-PI group who performed the test (mean ± SD) (71 ± 5.9% v 89 ± 7.23).

**Table 3:** Association between subjects with lung invovlement and the presence of other pulmonary complications within 2 years of follow-up, adjusted for age and gender

**Pulmonary complications**	**OR (95% CI)**	***P*-value**
Pulmonary hypertension	1.21 (0.92, 1.28)	0.082
Pulmonary fibrosis	1.79 (1.48, 2.12)	<0.001
Pulmonary infarction	0.99 (0.91, 1.11)	0.222
Pleural fibrosis	1.13 (0.94, 1.21)	0.138
Chronic asthma	1.27 (1.12, 1.38)	0.072
FEV1/FVC ratio*	1.42 (1.27, 1.84)	0.045
DLCO/VA**	1.31 (1.22, 1.76)	0.045

*Ratio of forced expiratory volume in one second (FEV1) to force vital capacity (FVC).

**Ratio of diffusing capacity of the lung for carbon monoxide (DLCO) to alveolar volume (VA).

## Discussion

AAV represents a very heterogeneous group of diseases with disease-specific and overlapping clinical features [6]. Although current therapies have reduced the rate of mortality, the quality of life remains poor. Therefore, recent studies to identify high-risk clinical features have enhanced categorization of AAV using accurate stratification for future therapeutic, epidemiological and basic research [4,13].

BVAS and VDI are clinical tools that may provide an accurate description of the current status of an individual patient in a standardized manner. Studies evaluating long-term prognosis and risk factors have reported organ-specific involvement as important indicators. GI involvement at initial presentation in subjects with CSS is associated with a high rate of relapse whilst the presences of renal disease and pANCA positivity are associated with lower rate of relapse [14]. Damage has been a major concern with regard to patient outcome in AAV. Since 1997, the VDI has been the only validated tool for damage in AAV [15].

The role of LI at initial presentation remains controversial [13]. A recent study with 80 subjects presenting with DAH in AAV showed that AAV was not associated with increased deaths compared to renal insufficiency [10]. In addition, ENT manifestations were associated with a better outcome in GPA [11]. In a study with 155 subjects with GPA that were followed-up for a median of 7 years, LI at diagnosis was predictive of a >3-fold higher mortality [8]. Another study showed that DAH in MPA was associated with 8.65 RR of mortality [7]. PI was also a predictor of survival with renal involvement. A later study by the same group showed that in 350 subjects with renal AAV, lung and ENT involvement were associated with RR of 1.7 for relapse [16]. The authors postulated that the likelihood of pulmonary infection and/or colonization is increased due to previous disease activities leading to structural damages such as cavitating lesions and further infection. This may lead to changes in vascular flow trauma and adaptations responsible for future relapses. External agents have been shown to increase relapse such as nasal colonization with S. aureus in GPA [17].

Our present study investigated the association of damage and disease activity with PI at initial presentation in subjects with AAV. There were no differences in BVAS3 and VDI between the PI and non-PI group. However, when the subjects were subcategorized into LI and non-LI groups, there were significant differences for both BVAS3 and VDI between the two groups at almost all time points. It is to be noted that discrepancy of symptoms existed in this study. 64% of subjects with PI were diagnosed with GPA. Interestingly, the overall upper respiratory tract or ENT involvement was less common at 20%. Tracheobronchial disease was also less common even though studies have reported up to 50-60% [18,19]. This is likely due to the design of the study which does not include subjects attending the ENT service due to isolated upper respiratory tract problems. In MPA, PI was not as common as GPA, however half of these were diagnosed radiologically in which radiographic infiltrates were the most common finding. We had a smaller number of subjects presenting with PI that were subsequently diagnosed with CSS. One of these subjects had minor infiltrates on the presenting chest radiograph. 2 subjects in the PI group were diagnosed with IPIPC in which one presented with limited alveolar hemorrhage. Therefore, we conclude that though discrepancy of symptoms existed in this cohort, the prevalence of different diseases represent the overall prevalence of these diseases in other AAV studies.

This is the first study evaluating BVAS3 in discriminating PI. The two main concerns flagged from BVAS version 2 were that two sub-scores were produced to reflect active and persistent disease respectively [20]. In BVAS3, subjects with purely persistent disease will generate a BVAS value on the same scale as those with ‘new/worse’ disease. The earlier BVAS2 has been shown to have prognostic value and correlates with the validated 5-factor prognostic score [21]. BVAS3 demonstrates concordance with BVAS2, treatment decision, physician global assessment and C-reactive protein [21], however, further studies are required to determine if BVAS3 correlates with mortality and/or health related quality of life. We have shown here that BVAS3 correlates with VDI. Accumulation of even a single VDI item is associated with reduced response to treatment and survival [22]. A 6-month VDI score >4 has been associated with increased risk of mortality [23].

We have not shown a correlation between PI and/or LI with risk of relapses or mortality. This provides additional data supporting recent studies looking at the low impact of LI on the overall AAV prognosis. However, the impact on the overall morbidity remained unanswered. Interestingly, the BVAS and VDI have been weakly correlated with patient health-related quality of life (HRQOL) reinforcing that quality of life cannot be generalized to biological effects of the disease only [24,25]. Future studies should examine the effects of LI in both physical and functional quality of life using objective assessment tools.

We showed a weak but statistically significant correlation between PI and the presence of musculoskeletal, renal and cardiovascular involvement within 2-years of follow up. Renal involvement was expected due to the high prevalence of pulmonary-renal syndrome in this cohort. Myalgia is included in the analysis of musculoskeletal involvement and therefore, may not impact on the overall prognosis. However, cardiovascular involvement was unexpected. This may relate to 4 and 6 subjects respectively reported to have pulmonary hypertension and coronary artery diseases in the PI group compared to 0 and 4 in the non-PI group respectively. Therefore, this correlation could be related to the differences in prevalence of coronary artery disease that may or may not be related to active vasculitis.

Interestingly, we also identified a significant relationship between pulmonary fibrosis and AAV. Although these parameters were recorded within 6 months to two years of follow-up, we cannot determine that pulmonary fibrosis is secondary to damage of previous lung involvement or a new, unusual manifestation of AAV itself. A few retrospective studies reported that pulmonary fibrosis is an underestimated manifestation of AAV, which often predates the development of vasculitis [26,27].

There are several limitations to this study. The overall number for specific diseases are small however, this study emphasized the long-term impact of LI in AAV as a single entity. Interestingly, there were differences in VDI when subjects were subcategorized into disease-specific populations, demonstrating that LI individuals with GPA and MPA had higher VDI compared to their counterparts. This observation could be mirrored due to the partly distinct clinical manifestations which may reflect the differences between active vasculitis and granulomatous inflammation. This reinforces the concept of AAV especially in regards to MPA and GPA that are a phenotypic continuum. Meanwhile, CS has distinct features related to atopy and eosinophilia despite shared features of pauci-immune glomerulonephritis and positive MPO-ANCA. AAV is recruited and those that were may be selected leading to bias. For example, as subjects were not recruited from all clinical services, other localized forms of AAV such as localized cardiac or intracranial vasculitis are not represented. Additionally, this study did not take into account the possible effects of different immunosuppressive regimens on damage scores and complications.

## Conclusions

Despite its limitations, this study is important as it highlights another issue in the natural history and prognosis of AAV. Although the outcome of AAV has improved significantly with the introduction of immunosuppressive therapy, subsequent disease course remains unsatisfactory for most subjects, due to low-grade active disease, relapse and the effects of damage due to established disease or drug toxicity. This study shows that subjects presenting with LI suffered considerable burden of disease which should drive the search to improve current management strategies in AAV. An important measure of future therapeutic approaches will be their ability to reduce the damage accrued over time.

## Abbreviations

AAV: ANCA-associated vasculitis; ANCA: Antineutrophilic cytoplasmic antibody; BVAS: Birmingham Vasculitis Activity Score; CI: Confidence interval; CS: Churg-Strauss; GPA: Granulomatosis with polyangiitis; LI: Lung involvement; MPA: Microscopic polyangiitis; MPO: Myeloperoxidase; OR: Odds ratio; PAN: Polyarteritis nodosa; PI: Pulmonary involvement; PR3: Proteinase 3; SD: Standard deviation; SEM: Standard error; VDI: Vasculitis Damage Index.

## Competing interests

The authors declare that they have no competing or financial interests.

## Authors’ contributions

TH – Data analysis, primary manuscript preparer, guarantor of the paper. AH and AI - Data collection. ML– Radiology analysis. CG – Data and statistical analysis. NGM – Data and manuscript reviewer. SJO – Direction of research, data and manuscript reviewer as senior author. All authors read and approved the final manuscript.

## Supplementary Material

Additional file 1: Figure S1 BVAS 3 scores at initial presentation. Data were compared by non-parametric *t-*test (Mann Whitney U) with a significance *p*-value cut-off of 0.05 (*p < 0.05, **p < 0.01, ***p < 0.001). Click here for file

## References

[B1] Little MA Nightingale P Verburgh CA Hauser T De Groot K Savage C Jayne D Harper L European Vasculitis Study (EUVAS) Group Early mortality in systemic vasculitis: relative contribution of adverse events and active vasculitis Ann Rheum Dis 2010 69 1036 1043 10.1136/ard.2009.109389 19574233

[B2] Walton EW Giant-cell granuloma of the respiratory tract (Wegener’s granulomatosis) Br Med J 1958 2 265 270 10.1136/bmj.2.5091.265 13560836 PMC2026251

[B3] Gordon M Luqmani RA Adu D Greaves I Richards N Michael J Emery P Howie AJ Bacon PA Relapses in patients with a systemic vasculitis Q J Med 1993 86 779 789 7906421

[B4] Mahr A Katsahian S Varet H Guillevin L Hagen EC Höglund P Merkel PA Pagnous C Rasmussen N Westman K Jayne D French Vasculitis Study Group (FVSG) and the European Vasculitis Society (EUVAS) Revisiting the classification of clinical phenotypes of antineutrophil cytoplasmic antibody-associated vasculitis: a cluster analysis Ann Rheum Dis 2013 72 1003 1010 10.1136/annrheumdis-2012-201750 22962314

[B5] Kamali S Erer B Artim-Esen B Gul A Ocal L Konice M Aral O Inanc M Predictors of damage and survival in patients with Wegener’s granulomatosis: analysis of 50 patients J Rheumatol 2010 37 374 378 10.3899/jrheum.090387 20008921

[B6] Frankel SK Schwarz MI The pulmonary vasculitides Am J Respir Crit Care Med 2012 186 216 224 10.1164/rccm.201203-0539CI 22679011

[B7] Hogan SL Nachman PH Wilkman AS Jennette JC Falk RJ Prognostic markers in patients with anti-neutrophil cytoplasmic autoantibody associated microscopic polyangiitis and glomerulonephritis J Am Soc Nephrol 1996 7 23 32 8808106 10.1681/ASN.V7123

[B8] Reinhold-Keller E Beuge N Latza U de Groot K Rudert H Nölle B Heller M Gross WL An interdisplinary approach to the care of patients with Wegener’s granulomatosis: long-term outcome in 155 patients Arthritis Rheum 2000 43 1021 1032 10.1002/1529-0131(200005)43:5<1021::AID-ANR10>3.0.CO;2-J 10817555

[B9] Zycinska K Wardyn KA Tyszko P Otto M Analysis of early death based on the prediction model in Wegener’s granulomatosis with pulmonary and renal involvement J Physiol Pharmacol 2007 58 Suppl 5 829 837 18204197

[B10] Kostianovsky A Hauser T Pagnoux C Cohen P Daugas E Mouthon L Miossec P Cordier JP Gueillevin L French Vasculitis Study Group (FVSG) Alveolar haemorrhage in ANCA associated vasculitides: 80 patients’ features and prognostic factors Clin Exp Rheumatol 2012 30 70 S77 S82 22640651

[B11] Guillevin L Pagnoux C Seror R Mahr A Mouthon L Le Toumelin P The Five Factor Score revisited: assessment of prognoses of systemic necrotizing vasculitides based on the French Vasculitis Study Group cohort Medicine (Baltimore) 2011 90 19 27 10.1097/MD.0b013e318205a4c6 21200183

[B12] Exley AR Bacon PA Clinical disease activity in systemic vasculitis Curr Opin Rheumatol 1996 8 12 18 10.1097/00002281-199601000-00002 8867533

[B13] Luqmani RA Flossmann O Outcome in small-vessel systemic vasculitis J Rheumatol 2006 33 1224 1227 16821262

[B14] Pavone L Grasselli C Chierici E Maggiore U Garini G Ronda N Manganelli P Pesci A Rioda WT Tumiati B Pavesi G VAglio A Buzio C Secondary and Primer Vasculitides Study Group Outcome and prognostic factors during the course of primary small-vessel vasculitdes J Rheumatol 2006 33 1299 1306 16783858

[B15] Bhamra K Luqmani R Damage assessment in ANCA-associated vasculitis Curr Rheumatol Rep 2012 14 494 500 10.1007/s11926-012-0291-1 22983618

[B16] Hogan SL Falk RJ Chin H Cai J Jennette CE Jennette JC Falk RJ Guillevin L Nachman PH Predictors of relapse and treatment resistant in antineutrophil cytoplasmic antibody-associated small-vessel vasculitis Ann Intern Med 2005 143 621 631 10.7326/0003-4819-143-9-200511010-00005 16263884

[B17] Stegeman CA Tervaert JW de Jong PE Kallenberg CG Trimethoprim-sulfamethoxazole for the prevention of relapses of Wegener’s granulomatosis. French Vasculitis Study Group N Engl J Med 1996 335 16 20 10.1056/NEJM199607043350103 8637536

[B18] Fauci AS Haynes BF Katz P Wolff SM Wegener’s granulomatosis: prospective clinical and therapeutic experience with 85 patients for 21 years Ann Intern Med 1983 98 76 85 10.7326/0003-4819-98-1-76 6336643

[B19] Anderson G Coles ET Crane M Douglas AC Gibbs AR Geddes DM Peel ET Wood JB Wegener’s granuloma. A series of 265 British cases seen between 1975 and 1985. A report by a sub-committee of the British Thoracic Society Research Committee Q J Med 1992 83 427 438 1448544

[B20] Mukhtyar C Lee R Brown D Carruthers D Dasgupta B Dubey S Flossmann O Hall C Hollywood J Jayne D Jones R Lanyon P Muir A Scott D Young L Luqmani RA Modification and validation of the Birmingham Vasculitis Activity Score (version 3) Ann Rheum Dis 2009 68 1827 1832 10.1136/ard.2008.101279 19054820

[B21] Gayraud M Guillevin L le Toumelin P Cohen P Lhote F Casassus P Jarrousse B French Vasculitis Study Group Long-term follow-up of polyarteritis nodosa, microsocopic polyangiitis and Churg-Strauss syndrome: analysis of four prospective trials including 278 patients Arthritis Rheum 2001 44 666 675 10.1002/1529-0131(200103)44:3<666::AID-ANR116>3.0.CO;2-A 11263782

[B22] Koldingsnes W Nossent JC Basline features and intial treatment as predictors of remission and relapse in Wegener’s granulomatosis J Rheumatol 2003 30 80 88 12508394

[B23] Exley AR Bacon PA Luqmani RA Kitas GD Carruthers DM Moots R Examination of disease severity in systemic vasculitis from the novel perspective of damage using the vasculitis damage index (VDI) Br J Rheumatol 1998 37 57 63 10.1093/rheumatology/37.1.57 9487252

[B24] Koutantji M Harrold E Lane SE Pearce S Watts RA Scott DG Investigation of quality of life, mood, pain, disability and disease status in primary systemic vasculitis Arthritis Rheum 2003 49 826 837 10.1002/art.11471 14673970

[B25] Basu N Jones GT Fluck N MacDonald AG Pang D Dospinescu P Reid DM Macfarlene GJ Fatigue: a principle contributor to impaired quality of life in ANCA-associated vasculitis Rheumatology (Oxford) 2010 49 1383 1390 10.1093/rheumatology/keq098 20400759 PMC3091420

[B26] Yamada H ANCA: associated lung fibrosis Semin Respir Crit Care Med 2011 32 322 327 10.1055/s-0031-1279828 21674417

[B27] Tzelepis GE Kokosi M Tzioufas A Toya SP Boki KA Zormpala A Moutsopoulos HM Prevalence and outcome of pulmonary fibrosis in microscopic polyangiitis Eur Respir J 2010 36 116 121 10.1183/09031936.00110109 19926741

