# Supplementary figures and images for: Lung involvement at presentation predicts disease activity and permanent organ damage at 6, 12 and 24 months follow - up in ANCA - associated vasculitis
Source: BMC Immunol. 2014 May 27;15:20. doi: 10.1186/1471-2172-15-20 (PMC4065082; doi:10.1186/1471-2172-15-20)

## Slide 1
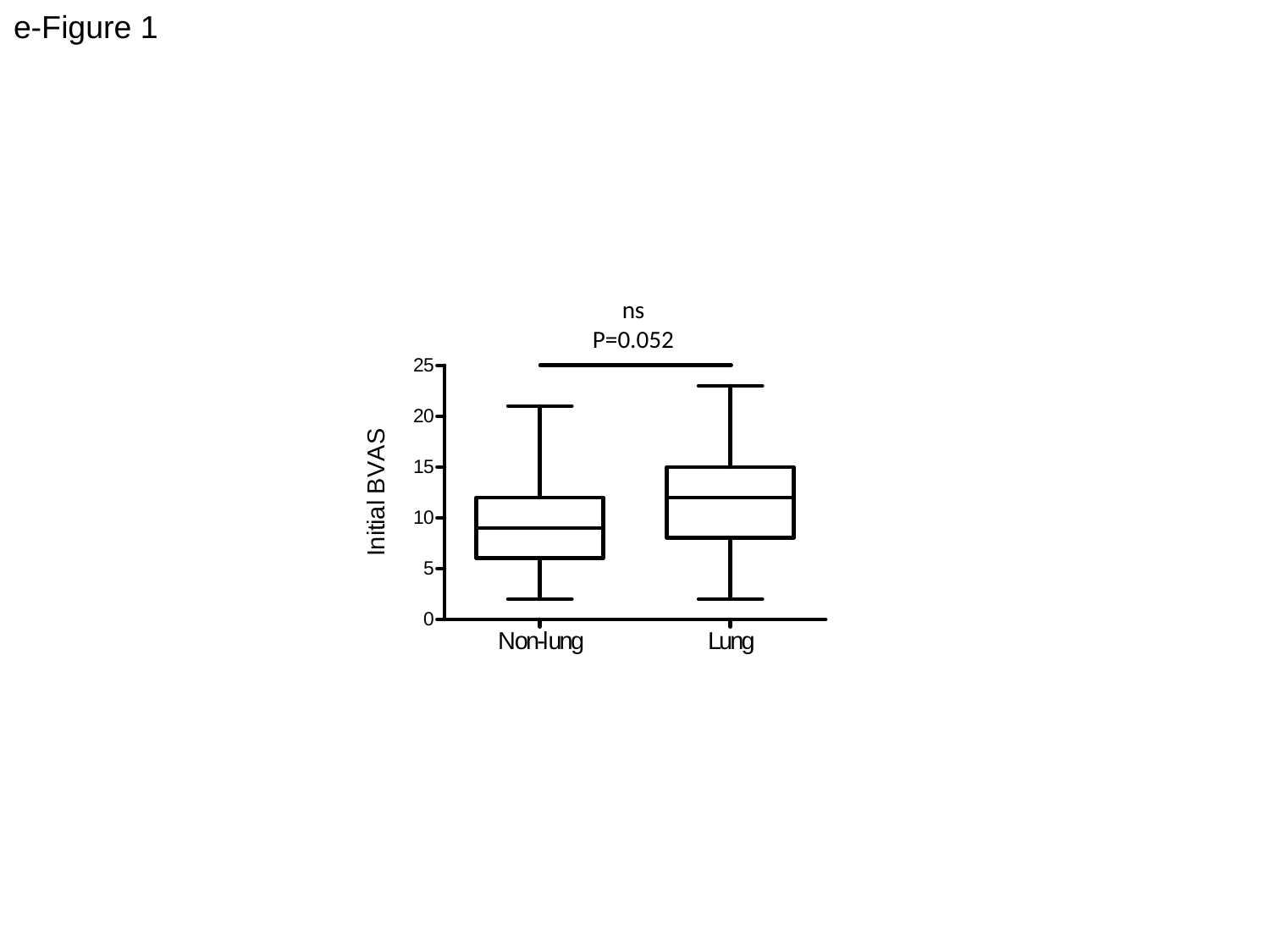

e-Figure 1
ns
P=0.052

Supplement: Additional file 1: Figure S1 — BVAS 3 scores at initial presentation. Data were compared by non-parametric t-test (Mann Whitney U) with a significance p-value cut-off of 0.05 (*p < 0.05, **p < 0.01, ***p < 0.001). [file 1471-2172-15-20-S1.pptx]
